# Pasteur and His Work

**Published:** 1886-04

**Authors:** 


### Art. VII.—PASTEUR AND HIS WORK.

The work of no other man in the realms of science, especially in the field of Preventive Medicine, has received so much criticism and so vigorous opposition as that of Pasteur. The reason for this must be sought in Pasteur himself, and not so much in his opponents. The "Life of Pasteur," which has been so frequently alluded to in the previous article, is in reality his life—the contradictions, the utter neglect of the work of others, past and contemporaneous—have been the very things most significant in Pasteur's methods, and which have called forth nearly all the opposition, and acted as a stimulus to scepticism. Pasteur is no chemist. Judging from what evidence we have at our command, he is not even a physiological chemist, nor has he any professional knowledge of the fundamental branches of medicine; and last, but most of all, we fail entirely in perceiving any evidence of a broad education in biology, zoology and philosophy which is absolutely necessary to a man's having comprehensive views in science.

After having done with his really great studies upon the beer, wine and vinegar ferments, and been of so much benefit to his country in almost saving its silk fabrication from destruction by studies of the disease of the silk worm, Pasteur next turned his attention to the study of some animal diseases. These have been anthrax, chicken cholera, hog cholera, and rabies. Pasteur did not approach either of them as a pathologist, other than that were contagious or infectious diseases, and caused great losses to France, or as in rabies, terrible sufferings to the few individuals who were unfortunate enough to be bitten by a rabid dog. Pasteur had no interest in either of these diseases. He did not care an iota about their nature other than their contagiousness, and so far as the nature of the pathological change occurring in them, they had no interest for him at all. To Pasteur they were simply a given something; that thing was nothing more or less than this: in the tissues of these animals there is lodged a specific quantity, this quantity is the contagious or infecting principle.

The next thing was what can be done with this principle? Pasteur is no bacteriologist; not one of his statements as to the existence of a specific form in a given disease are to be trusted unless we can find collateral support for them in the works of others. On the contrary, if we find such an assertion coming from his laboratory in Roux's name, we can depend upon it as if from a Koch or a Van Erningham, for Roux is a master in the technique of bacteriology, and the only micro-organism photographer in the world.

The idea of either an acquired or natural immunity from diseases of a certain character, is very, very old; hence Pasteur deserves no credit in that direction. In fact, unl ke such men as Darwin or Virchow, Pasteur is not a man of ideas, not a deep thinker; he will never move the world as a great teacher, but simply as the contributor of some new evidence; how much we do not yet know, of the fact first established by Jenner, that an artificial immunity is possible, by inoculating, what has been assumed to be another disease into the human organism to prevent a specific one.

In relation to small pox, the work of Pasteur goes most conclusively to show that the above statement is absolutely false, and that in reality we do not "inoculate another disease to prevent a specific one, as is generally believed, but that in reality the vaccina of the cow is nothing more or less than bovinated voriola humana; which has accidentally acquired a certain constancy of characters in the bovine species by direct yet accidental inoculation from cow to cow, until such a constancy has been acquired as Pasteur claims he has produced in rabbits with his spinal cord inoculations.

The only varieties of variola that have the least claim to isolated individuality, are the bovine and human. Whether they were ever connected we shall never know. History will not tell. The very next question, the easiest and most practical to treat, the question as to whether a virulent material containing the active principle in a given disease, can be attenuated by being passed through a serial number of given animals, and then finally acquire a constant yet attenuated virulence, capable of acting as a vaccine—is still—small pox.

It can be done at less expense and less danger than any other, and with the promise of the most satisfactory results.

Pasteur knew, as well as all know, of this acquired immunity from vaccination, as well as that acquired from persons having been through attacks of certain specific diseases.

The most interesting question to decide is, how did Pasteur approach this question of producing immunity; assuredly as a chemist! But what kind of a chemist? Did he approach it as an analytical or sympathetical chemist? Neither!

Pasteur went at this vaccine question in the most empirical manner it is possible to think of. He had a given something to do with, to him it was a virus. Knowing of Jenner's experiences he may have reasoned with regard to the vaccina, and it is probable he did, for we find him passing anthrax material serially through all sorts of animals. It is very probable that he was led to the idea of trying the effects of an exposure to high temperature on anthrax material, from his observations on hens—where he claims to have found that their normal temperature of 42° C., was opposed to their life, or virulence, but when he cooled the hens off that they died of anthrax. The idea of serial attention in animal inocculations was long before established by Rey at Lyons, but never came to any practical application.

Pasteur utterly ignores the work of others; to my mind the reason for this is to be sought in the fact that it is largely medical, and hence knowing nothing of it, it would be of little value to him again. He does not even quote or refer to the work done by others in bacteriology.

The reason for this may be sought in two things, he does not care much whether he knows the nature of the bacteria present or not, hence that matters nothing to him.

Certainly it simplifies matters to him to be able to have them always on hand from pure cultures, but it is not generally known that neither his anthrax nor hog cholera vaccines are made from such pure, artificial cultivations, but his bouillons are secured directly from the hearts blood of animals that have been previously profusely inoculated with germs or blood from others. It is enough for Pasteur that he has this so-called virus at hand; then he goes on trying the effects of heat and cold, air and no air, one animal inocculation and another, with all sorts of contradictory results, but ever with one purpose in view, and that is to find a satisfactory method of producing out of this known or unknown something, another known or unknown something that will prove by animal experiment to give a satisfactory result.

How can we criticise such a man? They are certainly very wide from what we call scientific methods, or the manner, other men work. They are beyond all criticism, yet open to any objection. No one can follow his work, or can it be exactly contested, for we can never tell whether we are following him exactly. If you take any one of his works we shall find such a mass of contradictory statements as can be found in the writings of no other man. We have no well-arranged and systematic record of experiments giving every data, and all his conclusions are carefully reconsidered, as we find in the work of nearly every other scientific observer of this century. We find a positive assertion of results to-day contradicted by equally positive ones a week hence, but all through it we find one fact, the one point aimed after becomes more and more confirmed.

Judged by the standard we would measure other men Pasteur is not a scientist, judged by the benefits which his endeavors will confer on mankind, Louis Pasteur is one of the greatest geniuses that has ever lived. No matter what objections may be made, the result of the most sceptical testings of his work on anthrax and hog cholera by the Germans have shown that there is a valuable truth at the bottom of Pasteur's claims—that his virus will prevent it—but under conditions.

While his anti-rabies virus seems, on the face of it, to be the most untrustworthy of all, I believe on the contrary it to be the most perfected of all Louis Pasteur's apparent crude and truly empirical methods. They are the results of patient, untiring devotion to an idea; they have been in part successful, and whatever else may result, Louis Pasteur has indicated a way in which we can work, open to all, that must eventually lead to the saving of millions of dollars to the national wealth, and no one does compute its influence on the fate of humanity.

